# Strength training intervention for adult individuals with knee osteoarthritis: Establishing fidelity

**DOI:** 10.3389/fphys.2025.1583153

**Published:** 2025-06-18

**Authors:** Hamza Küçük, Niloufar Ghadamyari, Fatma Neşe Şahin, Güner Çiçek, Tülay Ceylan, Özkan Güler, Onur Mutlu Yaşar, Cüneyt Şensoy, Cansel Arslanoğlu, Erol Doğan, Erkal Arslanoğlu

**Affiliations:** ^1^ Yasar Dogu Faculty of Sport Sciences, Ondokuz Mayis University, Samsun, Türkiye; ^2^ Department of Sports Health Science, Graduate School of Health Sciences, Ankara University, Ankara, Türkiye; ^3^ Department of Coaching Education, Faculty of Sport Sciences, Ankara University, Ankara, Türkiye; ^4^ Faculty of Sports Sciences, Hitit University, Çorum, Türkiye; ^5^ Graduate School of Education, Ondokuz Mayis University, Samsun, Türkiye; ^6^ Faculty of Health Sciences, Izmir Demokrasi University, Izmir, Türkiye; ^7^ Pakistan Embassy International Study School, Ankara, Türkiye; ^8^ Faculty of Sport Sciences, Sinop University, Sinop, Türkiye

**Keywords:** adult individuals, osteoarthritis, physical performance, resistance training, strength exercise

## Abstract

**Background:**

Knee osteoarthritis (KOA) is a prevalent chronic condition among the adult individuals, leading to pain, joint stiffness, and muscle weakness. Resistance training is an effective strategy for alleviating KOA-related symptoms and improving physical function. However, the efficacy of such interventions also depends on their fidelity, ensuring that the prescribed exercise protocols are followed correctly. This study aimed to assess the fidelity of a structured strength training program and its effects on pain management in adult individuals with KOA.

**Methods:**

A total of 72 adults (mean age = 56.27 ± 4.89 years), approximately 40% of whom were overweight, were randomly assigned to either a strength training group (n = 37) or a control group (n = 35). Quantitative data were collected using the Knee Injury and Osteoarthritis Outcome Score (KOOS), while qualitative data on intervention fidelity were obtained through self-reported adherence to daily home workouts.

**Results:**

The results indicated a significant improvement in KOOS scores in the strength training group compared to the control group (p < 0.001). Participants consistently adhere to the prescribed exercise program in their home workouts. These findings highlight the importance of intervention fidelity in strength training programs for adult individuals with KOA, emphasizing its role in optimizing health outcomes.

**Conclusion:**

This study contributes to the growing evidence supporting structured strength training as a viable strategy for managing KOA-related symptoms and enhancing physical function in ageing populations.

## 1 Introduction

Knee osteoarthritis (KOA) is a group of chronic joint diseases associated with decreased muscle mass and increased pain (Sim et al., 2020). About two hundred fifty million people worldwide and two hundred forty-nine thousand people in China, suffer from KOA, an increase of 128.7% in China since 1990 (Postler et al., 2018; Gao et al., 2024). About 19.4% of Chinese people living with KOA are older than 60 years (Xiang and Dai, 2009); with a higher incidence rate in women (10.3%) than men (5.7%) (Tang et al., 2016). Research in China shows that with population ageing, KOA will significantly increase disability and societal costs among the adults (Cai et al., 2021). Osteoarthritis represents an escalating global health burden, with incidence rates rising across diverse populations from 1990 to 2019, mainly due to demographic shifts such as ageing and population growth. The increasing prevalence in various countries and the widening disparities between socio-demographic groups highlight the urgent need for targeted public health interventions and equitable healthcare resource allocation (Cao et al., 2024).

Osteoarthritis is the most common joint disorder and a cause of disability in this population group, which can affect people’s quality of life by causing pain, reducing physical performance, and thus limiting individual independence (Lu et al., 2015). This complication is a type of non-inflammatory joint disease that manifests in mobile joints by destroying articular cartilage and new bone formation on the surface and margins of the involved joints.

Osteoarthritis treatment includes pharmacological and non-pharmacological approaches and mainly focuses on reducing pain and improving physical function and quality of life (Brown, 2020). One of the main components of osteoarthritis treatment is strengthening the function and strength of the muscles around the joints because muscle weakness makes the joint more susceptible to damage (Ahanjan et al., 2013). Physical activity as a non-pharmacological and non-surgical measure, can restore the physiological function of synoviocytes, prevent osteoarthritis and postpone the need for joint replacement (Di et al., 2019; Simão et al., 2019; Caiado et al., 2022; Santos et al., 2024).

The results of a systematic review in 2021 showed that implementing exercise programs in patients with knee osteoarthritis is safe, and effective, and mainly improves pain (Raposo et al., 2021). In addition, physical activity and exercise are interventions that have few side effects and can improve pain intensity, physical function, and thus, quality of life, thereby improving overall physical and mental health (Geneen et al., 2017; Santos et al., 2024). Exercise programs that strengthen the hip and leg joints reduce pain in patients with KOA (Lun et al., 2015). The effects of pain relief with exercise therapy are not only more significant than non-steroidal anti-inflammatory drugs and acetaminophen but do not involve the risk of side effects of drugs (Skou et al., 2018).

Within adult population, there is a growing need to manage pain caused by KOA and to find ways to increase the effectiveness of strength exercise programs. It is, therefore, important that adequate intervention fidelity is developed to manage pain in the adult individuals. Intervention fidelity is one of the main elements in designing a training program and is implemented to deliver an intervention study competently (Bond et al., 2022). Intervention fidelity considers a variety of variables necessary to deliver an intervention, so we will know if the intervention is delivered as designed.

Recent research (Marcos-Frutos et al., 2025) has looked into the specific contributions of strength training. People can opt for either weight-based or non-weight strength training methods according to their personal preferences, as the effects and adaptations from using machines for strengthening the quadriceps have been found to be quite similar to those from weight-free strength training for the same muscle. As a result, we view the research (Zhang, et al., 2025) as consistent with the current study and believe that non-weight-bearing strength exercises can play a specific role in rehabilitation for alleviating pain in individuals with knee osteoarthritis. Additionally, patients may choose to engage in non-weight-bearing strength exercises to reduce knee pain based on their personal preferences. In accordance with the beneficial impacts of strength training on enhancing functional capacity, research conducted by (Güney et al., 2024) analyzed the influence of strength training on shoulder muscle strength between a group engaged in digital gaming and one that was not. The findings indicated that strength training boosts both functional ability and the motor capacity of the targeted muscles. Consequently, for individuals with knee osteoarthritis, emphasizing the efficacy of the quadriceps muscle’s functional abilities suggests that this efficacy can enhance the functionality of the muscles surrounding the knee joint and improve these patients' quality of life.

Intervention fidelity is a methodological strategy with at least five major components, which should be theoretically separated (Gearing et al., 2011; Yates et al., 2013; Kechter et al., 2019): (a) study design, ensuring the avoidance of cross-contamination between study groups; (b) provider training, ensuring the standardization of provider training; (c) treatment delivery, ensuring the delivery of the treatment as designed; (d) treatment receipt, ensuring the actual reception of the treatment by the participant; and (e) treatment enactment, ensuring the measurement of the actual participation of subjects in the program. It has been shown that in several studies, the treatment fidelity practices of study design, provider training, and treatment delivery are related to therapists and trainers (Kechter et al., 2019). Furthermore, the practices of receipt and enactment seem to be a serious issue for participants as they are related to intervention recipients (Borrelli, 2011).

Whereas receipt and enactment are widely recommended for evaluating the intervention fidelity (Rixon, et al., 2016; Miles et al., 2023), research concerning the treatment fidelity assessment of receipt and enactment is scarce. Benefits of assessing intervention fidelity by asking the intervention recipients include a reflection on the understanding, usage, and application of the skills taught in their daily activities (Rixon, et al., 2016). Since the intervention recipients approach has received little attention in studies evaluating intervention fidelity (Breitenstein et al., 2010; Begum et al., 2021), the methods used by intervention recipients to evaluate intervention fidelity are mainly unknown. Participation in a structured strength exercise training program will significantly reduce pain levels in adults with knee osteoarthritis (KOA), and this effect will be mediated by two key fidelity components: receipt (understanding of the intervention) and enactment (actual implementation of the exercises). This study evaluates the impact of a strength exercise training program on pain and explores how two mechanisms, receipt, and enactment, may drive changes in the pain of adult patients with KOA.

## 2 Material and methods

### 2.1 Study design

This study was undertaken as a randomized controlled trial combined with quantitative and qualitative methods. This mixed methods study compared an intervention group and a usual care control group. At the beginning of the study, the KOOS score was administered to the groups. After a period of 4 weeks, the KOOS score was administered to the participants again. Then, qualitative measures were conducted with the strength training group to assess their adherence to the strength training intervention.

The study was conducted on a home-compiled program at Urmia Milad International Hospital and was approved by the Regional Clinical Ethics Review Board in Urmia with the number IRCT20230128880092N6.

### 2.2 Participants and procedures

A total of 72 adults (mean age = 56.27 ± 4.89 years), approximately 40% of whom were overweight, were randomly assigned to either a strength training group (n = 37) or a control group (n = 35). G*power 3.1.9.2 software (Germany) was employed to determine the sample size. Thus, 30 participants were enough based on the effect size of 0.60, test power of 0.95, and significance level of 0.05. The sample consisted of all consecutively admitted elderly patients with KOA at the hospital between February 2021 and March 2023. Eligible inclusion criteria for joining the trial in both groups were: Elderly in both groups were eligible to join the trial if they met the following inclusion criteria: (1) Having the American College of Rheumatology (ACR) criteria clinically and radiographically that according to these criteria, KOA is defined as having knee pain and having three criteria like age over 38 years, morning stiffness less than 30 min, and crepitus; (2) experiencing knee pain for most days of the last month; (3) walking and doing daily activities independently; (4) physical and mental ability to complete questionnaires; (5) participating in sports exercises based on the diagnosis of the attending physician; (6) blood pressure stability. The exclusion criteria of the research were: (1) Elderly with symptoms such as knee locking; knee joint surgery, and knee replacement during the last 6 months. (2) clarify deformation of the knee (3) knee malignancy history; (3) excessive knee movement and ligament instability; (4) the existence of metabolic and endocrine disorders such as diabetes and thyroid diseases; (5) having health problems affecting home exercises such as uncontrolled high blood pressure, heart disorder, brain disorder, shortness of breath, and arrhythmia. The adult who had these symptoms were excluded from both groups.

Participants in the strength exercise group performed the exercises taught, while participants in the control group received usual care, including the prescribed use of medications, and observation of the correct technique for using knee joints.

**FIGURE 1:**
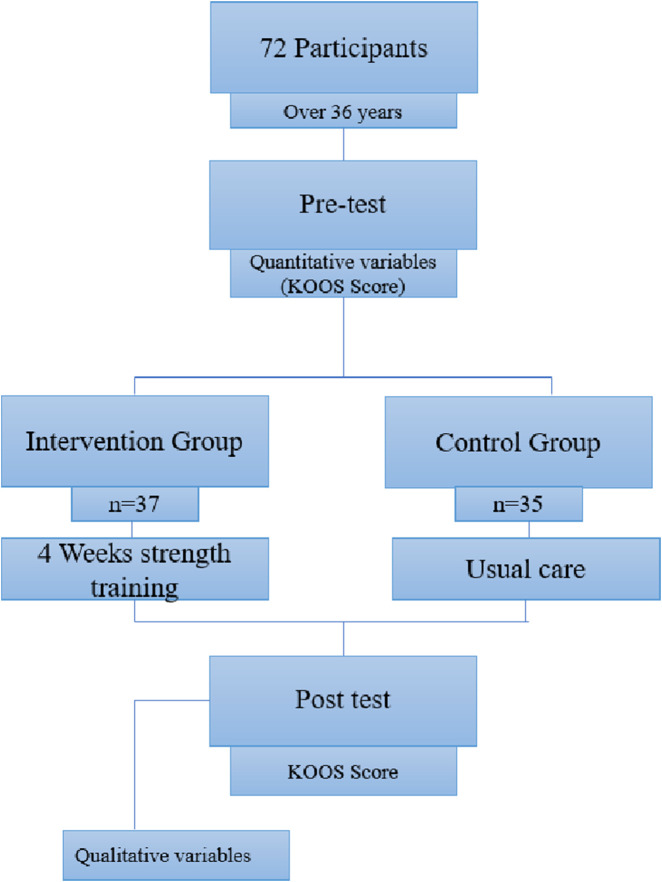
Flow diagram of the study design.

Assessments were conducted online (baseline and after intervention) for the intervention group and the control group. Quantitative variables were assessed at two time-points. The qualitative variable (weekly homework) was assessed by the intervention group after the intervention (Figure 1).

**FIGURE 2:**
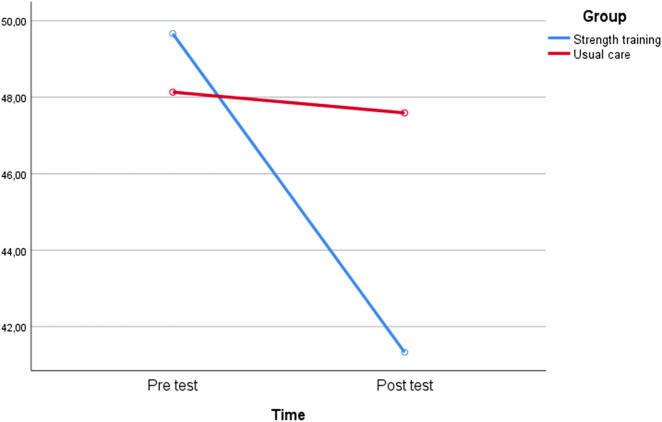
Graph of time-dependent change.

### 2.3 Strength exercise program

The study developed the training program with respect to another strength exercise training program, which has been shown to have a positive effect on pain in the adult individuals with KOA (Safarnia et al., 2022). First, during a period of 4 weeks, the patients in the intervention group were trained during strength sessions by the researcher in the clinic hall of the Milad hospital, on how to do sports exercises as follows (Table 1). Then, the patients performed the taught exercises for 1 month at home according to the written program.

**TABLE 1:** Strength training process.

Sessions	Exercises
1	Seven exercises to strengthen quadriceps muscles
2	Two exercises to strengthen the gluteus maximus and maintain balance while standing and walking
3	Three exercises to strengthen the legs
4	Four exercises to strengthen legs, one exercise to strengthen the muscles of the side, stomach and thigh, and two exercises to strengthen inner thigh muscles

The researcher communicated with the patients via message and phone to ensure that the daily exercises were performed correctly and on time. In addition, the adult or a family member informed the researcher how to perform the exercises by sending pictures or videos, and the main researcher followed the treatment process for 1 month.

### 2.4 Quantitative measures

Sociodemographic and KOA-related variables. Data regarding the gender, age, height, weight, body mass index, history of using stairs, frequent heavy load carrying, sitting on all fours, joint trauma, and family history of diseases common in the adult individuals were obtained.

Fidelity of the strength exercise intervention. “Knee injury and Osteoarthritis Outcome Score (KOOS)” (Roos et al., 1998) was used to measure pain intensity. It contains 42 items divided into five subscales: knee pain intensity, severity of other disease symptoms, difficulty in performing daily life activities, difficulty in performing sports-recreational activities, and quality of life related to knee problems. In this questionnaire, pain intensity was measured with nine items. The items are rated on a five-point Likert-type scale as in the original KOOS: 0 (nothing), 1 (low), 2 (moderate), three (intense), and four (extremely intense); total scores range from 0 to 100. In the present study, internal consistency was good to excellent, with a Cronbach’s alpha range over time of 0.86–0.92.

The qualitative measure used to evaluate the fidelity of the strength exercise intervention was the weekly homework recordings of the adult individuals with KOA who participated in the strength exercise training program. The weekly homework assessed whether the older adults used the skills they were taught (Table 3).

### 2.5 Statistical analyses

Before and after the exercises, the patients were evaluated using the pain subscale of the knee injury and osteoarthritis outcome score (KOOS). Repeated Measures ANOVA (two-way) was used to compare the pre-test and post-test scores of the groups. Since the assumption of sphericity was violated according to Mauchly’s test, the Greenhouse-Geisser correction was applied. Bonferroni *post hoc* test was conducted to compare the main effects. The collected data were analyzed using SPSS 26 software.

## 3 Results

Tables 2, 3 show demographic and KOA-related variables of the adult individuals who participated in the study and those of the eligible non-participants who consented to participate. As the table shows for the intervention and control groups, the gender distribution was thirty-one male and forty-one female adult participants with KOA aged between 38 and 65 years, with a mean (SD) age of 56.27 (4.89) years. About 40% of the elderly had a body mass index (BMI) indicating they were overweight. In addition, about 50% of them had between 1 and 2 months of KOA illness. Furthermore, more than 60% had a history of using stairs, >70% had a family history of illness, and >60% had a history of injury to joints. As indicated in Table 2, there are significant improvement in KOOS scores in the strength training group compared to the control group (p < 0.001).

**TABLE 2:** Comparison of exercise and usual care groups.

Group		Pre-test		Post test					
	N	Mean	SD	Mean	SD	Time	Partial Eta	Time*Group	Partial Eta
Strength training	37	49.66	8.95	41.33	6.18	<0.001	0.197	0.001	0.159
Usual care	35	48.13	6.58	47.59	5.68				

According to the obtained results, there is a statistical difference between time (p < 0.001) and groups (p = 0.001).

**TABLE 3:** Adult Individuals' use of strength exercise skills reported in their weekly homework.

Strength exercise skills	Adults with homework completed (n)
Transform irregular exercises into strength exercises (week 1)	37
Highlight positive aspects of the exercises (week 1)	37
Interrupt irregular exercises by integrated pre-set programs (week 2)	37
Note the need to practice strength exercises (week 2)	37
Initiate integrated exercises with each part (week 2)	37
Nurture ways to challenge irregular exercises (week 3)	37
Generate positive activities by controlling irregular exercises (week 3)	37

In order to monitor and ensure that exercises were performed by the intervention exercise group and qualitative data obtaining from the weekly homework included the following four questions: 1) Dear participant, can you explain the strength exercise strategy learned this week? 2) Dear participant, did you do the exercises for this week at home? Please explain in detail. 3) Dear participant, could you apply the exercises you learned daily? Please explain in detail. 4) Dear participant, do you feel that the session was helpful for you?

As you can be seen in Table 3, all adults in the strength exercise group (n = 37) indicated that the program was informative (n = 37/37), comprehensible (n = 37/37) and learnable (n = 37/37). Adults who described the use of strength exercise skills in their weekly homework (n = 37). completed all their weekly homework). All participants reported that applying and retaining the learned skills needs more time. (n = 3/37). Also, participants indicated that the situation improved by the program, no one indicated that the situation worsened by the program and all participants indicated that their problems have decreased by the program. About helping to better learn the strength exercise intervention, participants provided great suggestions such as having more examples (n = 37), a transcript (n = 37), printouts (n = 37), or a written material (n = 37). Moreover, participants (n = 37) indicated that they did not feel the need.

## 4 Discussion

The increasing prevalence of knee osteoarthritis (KOA) and its associated pain symptoms have made effective non-pharmacological interventions a priority in clinical practice. The present study evaluated the implementation fidelity of a strength exercise program to help the adult individuals with KOA relieve their pain intensity. According to the international recommendations for the diagnosis and treatment of KOA, exercise therapy is currently accepted as a first-line treatment approach (Bannuru et al., 2019). The program focused on developing seven strength exercise capabilities that are most important to reducing pain intensity.

The findings of this controlled trial provide evidence that the strength exercise program significantly reduced the pain intensity of the adult individuals with KOA over 4 weeks (Figure 2). Knee osteoarthritis (KOA) adult individuals in the strength exercise group reported improved pain scores post-intervention, indicating a long-term positive effect of the program on pain in this sample. In contrast, the usual care group showed minor positive effects of decreased pain. Therefore, the strength exercise program supported the control of pain intensity in these adults with KOA. Prior reviews and meta-analyses have shown that exercise helps individuals with KOA perform better and have less pain (Goh et al., 2019; Moura-Fernandes et al., 2020; Moreira-Marconi et al., 2020; Raposo et al., 2021; Choi et al., 2025). Current research in line with previous studies (Nguyen et al., 2016; Verhagen et al., 2019) showed that resistance training is a popular rehabilitative exercise modality that has been widely used to treat a variety of musculoskeletal conditions. Among these, this research found that resistance training successfully relieves pain, improves functional ability, and increases muscle strength. However, comparisons of the effects of weight training with aerobic workouts, aquatic exercises, and other types are still debatable (Jiang et al., 2024). Similar research results showed that balance exercises and yoga can also be effective in reducing muscle pain and stiffness, and improving athletic performance and sleep (Cheung et al., 2014). However, studies that contradict the present study have shown that acute resistance exercise can lead to increased pain sensitivity and decreased pain threshold in KOA patients (Burrows et al., 2014). It has been discovered that therapeutic exercises, for instance, isotonic, isokinetic, and isometric exercises, help individuals with KOA feel less pain (Kangeswari et al., 2021). Consistent with previous studies that found the positive effects of isometric and isotonic exercises on pain (Altaş and Demirdal, 2020) and the aquatic exercise program on knee pain and stiffness (Kars Fertelli et al., 2019) in KOA patients, this study showed the long-term effects of the strength exercise program.

In the current study, we developed two aspects of intervention fidelity (receipt of the intervention and enactment by intervention recipients) to drive changes in the pain of adult patients with KOA. Our results showed that these outcomes significantly improved strength exercise skills for the adult individuals with KOA in the strength exercise group. This is an important finding, consistent with the intended targets of effective teaching. According to new research today, exercising and walking by patients with KOA themselves can have many protective factors against KOA, and can even be effective in preventing and managing KOA (Wang et al., 2024). In line with recent research, the current study’s results suggest that adult patients with KOA in the strength exercise group could learn strength exercise skills and control their pain (Rixon et al., 2016; Miles et al., 2023). Additionally, patients are now more frequently seen as active contributors to healthcare rather than passive users, especially since self-management support for chronic diseases became available (Lorig and Holman, 2003). The active role demands patients to completely engage with, understand, and gain intervention-related skills in order to apply them in their daily lives (i.e., enactment) (Miles et al., 2023).

Due to the need of the KOA patients' research community for long-term exercise interventions (Sharma et al., 2024), we attempted to investigate the effects of strength exercise interventions in this study. We performed and implemented a strength exercise protocol, especially the resistance exercises listed in Table 1. Over 4 weeks, the intervention group was trained by the researcher during strength sessions in the clinic hall of the Milad hospital on how to perform sports exercises as follows. The patients then performed the taught exercises for 1 month at home, according to the written program that was given to them, to reduce their pain.

In contrast to other strength exercise interventions explored in previous studies, we measured the intervention fidelity from the adult individuals’ perspectives, to provide insightful comments regarding improving the intervention. As suggested by the KOA adult patients, future studies can include transcripts, printouts, or written material to help participants understand the strength exercise intervention better. Furthermore, additional studies (Jukic et al., 2020; Jukic et al., 2021) have shown the benefits of strength training, such as the fortification of cartilage, ligaments, and connective tissues around the knee joint, which may aid in alleviating pain and enhancing the functionality of the targeted muscle in individuals with knee osteoarthritis. Nonetheless, to fully realize the advantages of strength training, the optimal recommendations for different factors such as intensity, type, speed, and volume of exercise in healthy individuals continues to be debated. It is important to highlight that the current study aligns with earlier findings (Faulkner, 2012) on the necessity for innovative approaches, treatment delivery methods, and implementation strategies to evaluate and enhance adherence to exercise programs.

Future studies should also provide more concrete examples. In addition to these suggestions, a webinar with more interactions from the perspectives of KOA elderly patients should be included. From the perspectives of the five adult individuals, KOA adult patients might enjoy online classrooms and ask questions, leading to less stress. Therefore, a comparative study on online strength exercise training versus in-person strength exercise intervention is an important direction for future studies.

We focused on receipt and enactment as integral components of the strength exercise intervention fidelity from the viewpoint of the intervention recipients. At the end of the study, the responses given by the strength training group to the qualitative questions demonstrated the feasibility of the study. Besides these components, the impact of study design, provider training, and intervention delivery on the receipt and enactment of the strength exercise intervention among KOA adult patients should be examined to support its reliability and validity more fully.

The first limitation of this study is that the KOA adult individuals participated in in-person classrooms, while some participants wanted to continue in online classrooms. Therefore, the sample consists of KOA adult patients who could not use the internet. Another limitation is a sample size of participants in group. Most adult individuals had minor to moderate knee osteoarthritis and short hospital stays. Therefore, the sample may not represent the adult individuals with KOA in general or those with larger KOA involving lengthy hospital stays. Moreover, the strength of this study is that increasing muscle strength and improving functional ability in adult individuals through strength training interventions can help improve the quality of life of these patients with its long-term effect.

## 5 Conclusion

In this study, we used a unique approach: randomization, a usual care group, and KOA adult patients to examine the effectiveness of a strength exercise program on pain intensity. Nevertheless, some limitations should be noted. Our results show that intervention receipt and enactment can positively affect the fidelity of strength exercise interventions and can be used as an effective strategy to establish fidelity. Future research directions may also be highlighted. Although interventions in physical exercises for managing pain caused by KOA are increasing annually, there are many research questions concerning the effectiveness of such interventions. Concerning strength exercise programs, it is necessary to establish fidelity.

## Data Availability

The raw data supporting the conclusions of this article will be made available by the authors, without undue reservation.
